# Young adults’ self-sufficiency in daily life: the relationship with contextual factors and health indicators

**DOI:** 10.1186/s40359-020-00434-0

**Published:** 2020-08-28

**Authors:** Suzanne J. van den Toren, Amy van Grieken, Marlou L. A. de Kroon, Wico C. Mulder, Yvonne T. M. Vanneste, Hein Raat

**Affiliations:** 1 Department of Public Health, Erasmus University Medical Center, P.O. Box 2040, 3000 CA Rotterdam, the Netherlands; 2 Department of Health Sciences, University Medical Center Groningen, Groningen, the Netherlands; 3 Dutch Center for Youth Health (NCJ), Utrecht, the Netherlands

**Keywords:** Self-sufficiency, Young adults’ functioning in daily life, Mental health, Physical health, Finances, Health risk behaviors

## Abstract

**Background:**

Certain factors, such as depressive symptoms and binge drinking, may be linked to young adults’ ability to attain an acceptable level of functioning on specific life-domains (i.e. self-sufficiency). We studied the association of contextual factors and health indicators with self-sufficiency in young adults.

**Methods:**

We used both baseline (*n* = 755) and 6-months follow-up (*n* = 200) self-reported questionnaire data of intermediate vocational education students (16–26 years). The questionnaire included the adapted Dutch self-sufficiency matrix (SSM-D), which addresses self-sufficiency regarding 11 life-domains (e.g. finances and housing). The questionnaire also included potentially associated contextual factors (e.g. socio-demographic characteristics) and health indicators (e.g. sickness absence from school). Ordinal (overall self-sufficiency: self-sufficient on 11, 10, 9 or ≤ 8 life-domains), and logistic (self-sufficiency per life-domain: self-sufficient yes/no) regression models were applied.

**Results:**

The studied population was 18.6 years on average (SD 2.04), and 73.6% were female. Cannabis use was associated with a lower overall self-sufficiency category at baseline (OR = 0.57, 95% CI = 0.33–0.99), as were an increase in sick days (OR = 0.94, 95% CI = 0.91–0.98) and an increase on the scale of depressive symptoms (OR = 0.87, 95% CI = 0.85–0.89). An increase in sick days and an increase on the scale of depressive symptoms were associated with lower odds of being self-sufficient on three and ten life-domains, respectively (*p* < 0.05). An increase on the scale of depressive symptoms was associated with a lower overall self-sufficiency category 6-months post-baseline (OR = 0.90, 95% CI = 0.86–0.93).

**Conclusions:**

Our findings underline the importance of addressing self-sufficiency, sickness absence, and depressive symptoms, preferably before the transition from adolescence to young adulthood has begun.

## Background

Self-sufficiency is defined as the ability of individuals to attain an acceptable level of functioning regarding specific life-domains, such as daytime activities and social support. This ability could either be achieved by the person him/herself or by adequately organizing help from formal or informal care providers [1]. Enhancing self-sufficiency in emerging adults may contribute to a more successful transition from adolescence to adulthood [1, 2]. This transitional period is typically defined as a separate phase of emerging adulthood, a stage between adolescence and adulthood, which primarily exists in Western countries [3–5]. In this phase, adolescents transition from lower secondary school to further education or the labor market. Moreover, a transition occurs from dependence on parents to more autonomy and financial independence and from youth health care to adult health care. These transitions induce challenges for emerging adults in different life areas, e.g. finances, education and employment, leisure time activities, and physical and mental health behaviors [6–10]. These challenges might account for a decline in health status along with an increase in mental health problems and behaviors risky to health, such as binge drinking, smoking, and being physically inactive [7, 10–12]. Furthermore, the aforementioned transitions may result in a gap between the (health care) needs of emerging adults and the provision of care [10, 13, 14]. Therefore, studying self-sufficiency and potentially associated (risk) factors could inform the development of programs that aim to empower young adults’ functioning in daily life. Previous research illustrated that, for instance, financial self-sufficiency can be improved by financial education to students [15], and effective mental health services in the school context can help with a successful transition to adulthood [16].

Emerging adults attending intermediate vocational education (upper secondary education with specialized job-oriented programs, ISCED 3 [17]) are expected to struggle more with becoming self-sufficient than their peers from other school levels. These emerging adults display the highest percentage of leaving school without a diploma and report relatively high levels of risk behaviors, such as more than 50% of monthly binge drinking and 45% of daily smoking [18, 19].

This study examined the association of contextual factors (e.g. socio-demographics and context, and risk behaviors) and indicators of health status (e.g. sickness absence and depressive symptoms) with self-sufficiency in intermediate vocational education students aged 16–26 years, both cross-sectional and longitudinal. We hypothesized that less favorable contextual factors and less favorable indicators of health status will be associated with a lower likelihood of being self-sufficient.

## Methods

### Study design

For this study, baseline and follow-up data from the Medical Advice for Sick-reported Students (MASS) intervention evaluation study were used, which is described in more detail elsewhere [20].

The Medical Ethics Committee of the Erasmus University Medical Center Rotterdam reviewed the research proposal and declared that the Dutch Medical Research Involving Human Subjects Act (Dutch abbreviation: WMO) did not apply. They issued a declaration of no objection to conducting this study and permitted to submit the results for publication in a scientific journal in the future (proposal number MEC-2015-614). All participants provided written informed consent.

### Setting and study population

**Fig. 1:**
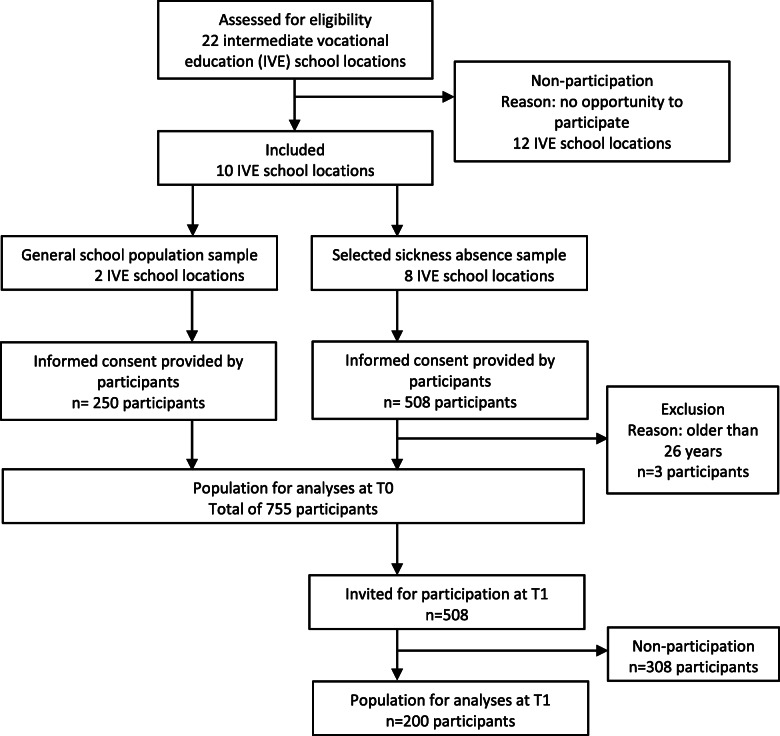
Flowchart of the participants in this study

A total of 22 intermediate vocational education school locations were invited to participate in the study (Fig. 1). Twelve locations could not participate, mainly because of the anticipated time investment. Finally, ten schools participated in the study in the Dutch regions of Utrecht, West-Brabant, Amsterdam, and Rotterdam. The baseline data was collected between December 2015 and October 2016. The follow-up data were collected between July 2016 and April 2017.

Participants were students aged 16–26 years attending intermediate vocational education level 1–4 (i.e. level 1 is considered assistant training and level 4 is considered middle management training). They attended the following vocational programs: media manager/developer, assistant care, trade, interior design, nursing, and technician studies. To adhere to the preferences of the schools, two different procedures were followed to select participants. (1) At eight schools, a school employee selected and invited students for participation if they had reported an extensive amount of sick days from school (i.e. reporting sick at least four times, or, more than six consecutive school days in twelve school weeks). (2).

At the two remaining schools, all students in randomly selected classes were invited to participate. The involved researcher selected students who met the criteria for extensive sickness absence afterward. All students from the first procedure and students from the second procedure who met the criteria for extensive sickness absence were placed in the ‘selected sickness absence’ sample. The remaining participants from the second procedure were placed in the ‘general school population’ sample (see Fig. 1).

All students and parents of students between 16 and 18 years of age were informed about the study through an information letter and a leaflet. These documents explained the aim of the study and included contact information of the researchers. An appointed coordinator from the participating schools sent the documents to the students and parents. Parents could object to having their child participate in the study by notifying their objection towards school or the researchers. The students were asked to provide written informed consent before filling out the questionnaire. The appointed coordinator collected all completed questionnaires and sent them to the researchers. Approximately 6 months later, the participants in the selected sickness absence sample (i.e. where the selection was based on the amount of sickness absence) received the follow-up questionnaire at home with a return envelope or through a link in their e-mail. The broader school sample was not invited to fill out the follow-up questionnaire. In total, 758 students provided written informed consent at baseline. Before analyses, we excluded students who were older than 26 years (*n* = 3).

### Measurements

The questionnaire contained the following topics: self-sufficiency, contextual factors (i.e. socio-demographic characteristics and context, and risk behaviors), and indicators of health status (i.e. sickness absence and depressive symptoms).

#### Self-sufficiency

An adapted version of the Dutch self-sufficiency matrix (SSM-D) was included in the questionnaire [1, 21]. This version was developed for and validated among students attending intermediate vocational education, corresponding to the language skills of the students, and addresses the ability of students to provide for themselves regarding 11 specific life-domains (finances, daytime activities, housing, domestic relations, mental health, physical health, addiction, activities daily life, social network, community participation, and judicial) [21]. Each life-domain was assessed by how many problems the student had in the past 6 months in a certain life-domain, e.g. ‘Finances. Think of: having the money to make ends meet’ (Additional file 1: Table A1). Five response categories ranged from ‘no problems’ to ‘many problems’. Response categories were dichotomized into ‘self-sufficient’ (few problems and no problems) versus ‘not to barely self-sufficient’ (many problems to not few/not many problems) for each life-domain. For analyses purposes, one overall self-sufficiency score was calculated, ranging from self-sufficient on all life-domains, ten life-domains, nine life-domains, and eight or fewer life-domains.

#### Contextual factors and indicators of health status

Elements of the International Classification of Functioning, Disability, and Health (ICF) from the World Health Organization were used to select relevant factors [22, 23]. In this framework, human functioning is considered at the level of the whole person in a social context. We applied an adapted version of this framework to our study (Additional file 2: Figure B1).

#### Contextual factors

*Socio-demographics and context* included age (years), gender (boy/girl), level of intermediate vocational education (higher level 4 vs. lower levels 1–3), ethnic background (Dutch versus non-Dutch by following the definition of Statistics Netherlands [24]), living situation, and perceived school performance. For living situation, ten different response categories were dichotomized into ‘living with a caretaker’ versus ‘living without a caretaker’. Perceived school performance was assessed by the question: “How do you think your teacher estimates your school performance compared to your classmates?”. Five response categories ranged from ‘very good’ to ‘not good’, and were dichotomized into ‘good’ versus ‘average or less’. Previous research showed this item can distinguish students who get good grades at school from students that do not [25].

*Risk behaviors* included cigarette smoking, binge drinking, cannabis use, delinquency, and truancy. Cigarette smoking was assessed by the question: “How often do you currently smoke?”. Four response categories ranged from ‘not’ to ‘yes, daily’ and were dichotomized into ‘current smoking’ versus ‘no current smoking’. Binge drinking was assessed by the question: “How many times did you consume five or more alcoholic drinks on one occasion in the past four weeks?”, by following the international definition of binge drinking [26]. Seven response categories ranged from ‘never’ to ‘nine or more times’ and were dichotomized into ‘not once’ versus ‘one or more times’. Cannabis use was assessed by the number of times the student had used cannabis over the past 4 weeks. Eight response categories ranged from ‘never’ to ‘20 or more times’ and were dichotomized into ‘not once’ versus ‘one or more times’. Criminal behavior was assessed by ten items covering criminal behavior in the past 6 months (e.g. bought stolen goods). Ten response categories ranged from ‘never’ to ‘six times’ or more. All ten items were merged into one dichotomous item ‘no criminal behavior’ versus ‘at least one criminal behavior’ in the past 6 months. Truancy was assessed by the question: “Have you been truanting in the past four weeks?”. Six response categories ranged from ‘no truancy’ to ‘more than 20 hours of truancy’, and were categorized into ‘never’, ‘1–5 h’ and ‘more than 5 hours’ [27].

#### Indicators of health status

Sickness absence was assessed by the question: “How many days in the past eight school weeks did you stay home from school, because you were sick? (do not count holidays)” [27]. The continuous score for number of sick days was used. Depressive symptoms were assessed using the validated Center for Epidemiologic Studies Depression scale (CES-D) [28, 29]. The CES-D is a 20-item scale used to determine the clinical relevance of depression. The items cover the main components of depressive symptoms such as depressed mood, guilt, feelings of helplessness, loss of appetite, and sleep. The frequency of experiencing these symptoms in the past week was assessed. Four response categories ranged from ‘always’ to ‘hardly ever’. The continuous total CES-D score was used with higher scores indicating higher levels of depressive symptoms (range of 0–60).

### Data analyses

Descriptive statistics were used to describe the socio-demographic characteristics of the study population (Table 1). We compared participants who completed both the baseline and follow-up questionnaire with participants lost to follow-up using chi-square tests (for categorical variables) and independent sample *t*-tests (for continuous variables) (Additional file 3: Table C1). Also, descriptive statistics were used to show the distribution of students who were self-sufficient and who were not self-sufficient in each life-domain at baseline and 6 months post-baseline (Table 2).

**Table 1:** Socio-demographic characteristics of the study population at baseline (*N* = 755)

Socio-demographic characteristics	Total population
**Age in years, mean (SD)**	
Age in years	18.6, range 16–26 (SD = 2.04)
**Gender, n (%)**	
Male	199 (26.4)
Female	554 (73.6)
**Intermediate vocational education, n (%)**^**a**^	
Level 1–3	249 (34.3)
Level 4	478 (65.7)
**Ethnic background, n (%)**	
Dutch	452 (60.6)
Non-Dutch	294 (39.4)
**Living situation, n (%)**	
With parents/caretakers	664 (88.1)
Not with parents/caretakers	90 (11.9)

Note: SD = Standard Deviation

^a^ Intermediate vocational education consists of four levels: 1 = assistant training; 2 = basic vocational training; 3 = vocational training; 4 = middle-management training

**Table 2:** Baseline and 6-months follow-up distribution of overall self-sufficiency and of the separate self-sufficiency life-domains

**Time-point**	**Overall self-sufficiency**			
	**On 11 life-domains** **n (%)**	**On 10 life-domains** **n (%)**	**On 9 life-domains** **n (%)**	**On ≤ 8 life-domains** **n (%)**
Baseline (*n *= 755)	265 (36.6)	116 (16.0)	103 (14.2)	240 (33.1)
Follow-up (*n* = 200)	58 (32.4)	38 (21.2)	20 (11.2)	63 (35.2)
	**Baseline (*n*** **= 755)**		**6-months follow-up (*n*** **= 200)**	
Finances	537 (73.5)	194 (26.5)	130 (72.2)	50 (27.8)
Daytime activities	545 (75.0)	182 (25.0)	147 (81.7)	33 (18.3)
Housing	661 (90.4)	70 (9.6)	171 (95.0)	9 (5.0)
Domestic relations	591 (80.8)	140 (19.2)	142 (78.9)	38 (21.1)
Mental health	437 (60.0)	291 (40.0)	97 (53.9)	83 (46.1)
Physical health	520 (71.1)	211 (28.9)	116 (64.4)	64 (35.6)
Addiction	663 (90.7)	68 (9.3)	168 (93.3)	12 (6.7)
Daily life skills	678 (92.7)	53 (7.3)	161 (89.4)	19 (10.6)
Social network	631 (86.3)	100 (13.7)	151 (83.9)	29 (16.1)
Community participation	566 (77.4)	165 (22.6)	142 (78.9)	38 (21.1)
Judicial	715 (97.8)	16 (2.2)	178 (98.9)	2 (1.1)

^a^ Self-sufficiency was measured on eleven life-domains. Participants indicated whether they were able to provide for themselves regarding these life-domains. Five response categories ranged from ‘no problems’ to ‘many problems’. Response categories were dichotomized into ‘self-sufficient’ versus ‘not to barely self-sufficient’ for each life-domain

We examined associations of contextual factors and indicators of health status (i.e. predictor variables) with self-sufficiency (i.e. outcome variable) using ordinal and logistic regression analyses. First, ordinal regression analyses were performed on baseline data to analyze the association between predictor variables and overall self-sufficiency at baseline as the ordinal outcome variable (ranging from self-sufficient on: all life-domains, ten life-domains, nine life-domains, and eight or fewer life-domains) (Table 3). Second, logistic regression analyses were performed on baseline data to assess the association between predictor variables and the separate self-sufficiency life-domains as outcome variables (Tables 4). Third, ordinal regression analyses were performed to analyze the association between baseline predictor variables and overall self-sufficiency at follow-up as the ordinal outcome variable (ranging from self-sufficient on: all life-domains, ten life-domains, nine life-domains, and eight or fewer life-domains) (Table 5). We also explored interaction between gender and all other predictors in the association between baseline predictors and baseline overall self-sufficiency. No significant interaction was found.

**Table 3:** Results of the cross-sectional ordinal regression analyses evaluating associations of contextual factors and indicators of health status with overall self-sufficiency (*n* = 755)

	Univariable model of self-sufficiency^a^	Multivariable model of self-sufficiency^b^
	OR (95%CI)*	OR (95%CI)*
**Contextual factors**		
*Socio-demographics and context*		
Age (in years)	**0.85 (0.80; 0.91)**	0.91 (0.83; 1.00)
Gender		
Male	Ref.	Ref.
Female	1.01 (0.75; 1.37)	1.09 (0.71; 1.65)
Intermediate vocational education^c^		
Level 1–3	Ref.	Ref.
Level 4	**0.62 (0.47; 0.83)**	0.72 (0.47; 1.09)
Ethnic background		
Dutch	Ref.	Ref.
Non-Dutch	1.28 (0.97; 1.69)	0.98 (0.66; 1.45)
Living situation		
With caretaker	Ref.	Ref.
Not with caretaker	**0.31 (0.20; 0.49)**	0.65 (0.34; 1.25)
Perceived school performance		
> Average	Ref.	Ref.
≤ Average	**0.39 (0.29; 0.52)**	0.79 (0.54; 1.15)
*Risk behaviors*		
Current smoking		
No	Ref.	Ref.
Yes	**0.60 (0.44; 0.80)**	0.81 (0.51; 1.27)
Binge drinking^d^		
0 times/ 4 weeks	Ref.	Ref.
≥ 1 time/4 weeks	**0.76 (0.58; 0.99)**	0.76 (0.51; 1.13)
Cannabis use		
0 times/4 weeks	Ref.	Ref.
≥ 1 time/4 weeks	**0.44 (0.31; 0.64)**	**0.57 (0.33; 0.99)**
Criminal behavior		
0 times/year	Ref.	Ref.
≥ 1 time/year	0.81 (0.55; 1.21)	1.19 (0.70; 2.02)
Truancy		
0 h	Ref.	Ref.
1–5 h	0.84 (0.62; 1.14)	0.95 (0.64; 1.41)
≥ 6 h	**0.37 (0.23; 0.59)**	0.59 (0.32; 1.11)
**Indicators of health status**		
Sickness absence (days/8 weeks)	**0.89 (0.86; 0.91)**	**0.94 (0.91; 0.98)**
Depressive symptoms (CES-D scale)	**0.87 (0.85; 0.88)**	**0.87 (0.85; 0.89)**

Note: Self-sufficiency was entered as an ordinal variable ranging from self-sufficient on all life-domains, self-sufficient on ten life-domains, self-sufficient on nine life-domains and self-sufficient on eight or fewer life-domains. Odds ratios represent the odds for a participant to be allocated within a higher self-sufficiency category if they would have scored one point higher on the predictor variable. Bold numbers indicate a statistically significant (*p* < 0.05) association

^*^Odds ratio (OR) and 95% confidence interval (95% CI) from ordinal regression analyses

^a^ The predictor variables were entered separately in the univariable model

^b^ The predictor variables were entered simultaneously in the multivariable model

^c^ Intermediate vocational education consists of four levels: 1 = assistant training; 2 = basic vocational training; 3 = vocational training; 4 = middle-management training

^d^ Binge drinking was defined as consuming five or more alcoholic drinks on one occasion

**Table 4:** Results of the logistic regression analyses evaluating associations of contextual factors and indicators of health status with separate self-sufficiency life-domains (*n*=755)

	**Finances**	**Daytime activities**	**Housing**	**Domestic relations**	**Mental health**	**Physical health**
	OR (95% CI)*	OR (95% CI)*	OR (95% CI)*	OR (95% CI)*	OR (95% CI)*	OR (95% CI)*
**Contextual factors**						
*Socio-demographics and context*						
Age (in years)	**0.84 (0.75; 0.94)**	0.96 (0.85; 1.09)	1.07 (0.89; 1.28)	0.98 (0.86; 1.12)	0.91 (0.79; 1.05)	0.92 (0.82; 1.03)
Gender (female)	1.05 (0.63; 1.74)	1.17 (0.69; 2.00)	1.15 (0.52; 2.52)	0.83 (0.45; 1.51)	0.73 (0.40; 1.34)	0.81 (0.48; 1.34)
Intermediate vocational education (level 4)^a^	0.78 (0.46; 1.34)	0.84 (0.49; 1.46)	1.03 (0.44; 2.40)	0.59 (0.32; 1.10)	0.57 (0.31; 1.06)	1.31 (0.80; 2.15)
Ethnic background (non-Dutch)	0.82 (0.50; 1.32)	0.83 (0.50; 1.38)	**0.47 (0.23; 0.98)**	0.62 (0.36; 1.07)	1.72 (0.98; 3.01)	1.33 (0.83; 2.13)
Living situation (not with caretaker)	**0.49 (0.25; 0.96)**	0.97 (0.45; 2.06)	**0.25 (0.10; 0.65)**	0.59 (0.27; 1.27)	1.50 (0.62; 3.65)	0.68 (0.34; 1.37)
Perceived school performance (≤ average)	0.86 (0.54; 1.39)	**0.37 (0.23; 0.59)**	0.73 (0.36; 1.49)	0.75 (0.44; 1.26)	0.72 (0.41; 1.25)	1.02 (0.64; 1.63)
*Risk behaviors*						
Current smoking (yes)	**0.49 (0.29; 0.84)**	0.91 (0.52; 1.60)	0.59 (0.25; 1.40)	**0.45 (0.24; 0.83)**	1.60 (0.85; 3.02)	0.81 (0.47; 1.37)
Binge drinking^b^ (≥ 1 time/4 weeks)	0.71 (0.43; 1.17)	**0.53 (0.32; 0.90)**	0.75 (0.34; 1.65)	0.96 (0.54; 1.69)	0.69 (0.39; 1.22)	1.11 (0.69; 1.78)
Cannabis use (≥ 1 time/4 weeks)	0.58 (0.33; 1.04)	0.73 (0.39; 1.38)	0.69 (0.28; 1.67)	0.88 (0.45; 1.72)	0.66 (0.31; 1.38)	0.71 (0.38; 1.32)
Criminal behavior (≥ 1 time/6 months)	0.69 (0.38; 1.25)	1.76 (0.88; 3.54)	2.09 (0.71; 6.18)	0.85 (0.43; 1.69)	1.16 (0.56; 2.42)	1.44 (0.75; 2.75)
Truancy						
0 hours	Ref.	Ref.	Ref.	Ref.	Ref.	Ref.
1-5 hours	1.00 (0.61; 1.64)	0.75 (0.45; 1.25)	0.74 (0.34; 1.62)	0.77 (0.45; 1.33)	0.73 (0.42; 1.26)	1.57 (0.96; 2.56)
≥ 6 hours	0.70 (0.36; 1.36)	0.57 (0.28; 1.15)	1.21 (0.41; 3.63)	1.37 (0.61; 3.08)	0.69 (0.30; 1.59)	1.93 (0.93; 4.02)
**Indicators of health status**						
Sickness absence (days/8 weeks)	1.02 (0.98; 1.05)	**0.92 (0.88; 0.95)**	0.96 (0.93; 1.00)	1.00 (0.97; 1.04)	0.96 (0.91; 1.01)	**0.92 (0.88; 0.95)**
Depressive symptoms (CES-D scale)	**0.96 (0.94; 0.98)**	**0.94 (0.92; 0.96)**	**0.93 (0.90; 0.96)**	**0.91 (0.89; 0.94)**	**0.81 (0.78; 0.84)**	**0.95 (0.93; 0.97)**
	**Addiction**	**Activities daily life**	**Social network**	**Community participation**	**Judicial**	**Total number of significant life-domains**
	OR (95% CI)*	OR (95% CI)*	OR (95% CI)*	OR (95% CI)*	OR (95% CI)*	**n life-domains**
**Contextual factors**						
*Socio-demographics and context*						
Age (in years)	1.11 (0.93; 1.33)	0.87 (0.72; 1.06)	0.96 (0.81; 1.13)	0.98 (0.87; 1.11)	1.23 (0.81; 1.86)	1
Gender (female)	**2.71 (1.34; 5.50)**	1.46 (0.58; 3.70)	1.40 (0.67; 2.91)	0.92 (0.53; 1.59)	**7.61 (1.20; 48.18)**	2
Intermediate vocational education (level 4)^a^	0.77 (0.35; 1.68)	1.55 (0.60; 4.03)	1.88 (0.90; 3.95)	**0.45 (0.25; 0.82)**	1.14 (0.21; 6.14)	1
Ethnic background (non-Dutch)	0.63 (0.32; 1.27)	1.12 (0.47; 2.69)	0.55 (0.28; 1.09)	0.66 (0.40; 1.09)	0.77 (0.15; 4.06)	1
Living situation (not with caretaker)	0.40 (0.16; 1.00)	0.81 (0.27; 2.46)	0.54 (0.21; 1.35)	0.71 (0.34; 1.47)	0.34 (0.03; 3.41)	2
Perceived school performance (≤ average)	0.75 (0.38; 1.45)	1.25 (0.54; 2.88)	0.77 (0.41; 1.46)	0.69 (0.43; 1.12)	1.18 (0.25; 5.56)	1
*Risk behaviors*						
Current smoking (yes)	0.52 (0.24; 1.14)	1.14 (0.41; 3.17)	0.81 (0.36; 1.84)	1.32 (0.73; 2.40)	1.79 (0.23; 13.72)	2
Binge drinking^b^ (≥ 1 time/4 weeks)	0.50 (0.23; 1.08)	0.73 (0.31; 1.76)	1.81 (0.88; 3.73)	0.76 (0.46; 1.28)	1.05 (0.17; 6.37)	1
Cannabis use (≥ 1 time/4 weeks)	0.59 (0.27; 1.28)	0.63 (0.22; 1.81)	0.78 (0.32; 1.94)	0.82 (0.42; 1.58)	0.78 (0.13; 4.68)	0
Criminal behavior (≥ 1 time/year)	1.03 (0.45; 2.37)	2.26 (0.56; 9.10)	**3.92 (1.22; 12.64)**	0.93 (0.47; 1.83)	**0.06 (0.01; 0.32)**	2
Truancy						
0 hours	Ref.	Ref.	Ref.	Ref.	Ref.	
1-5 hours	0.72 (0.35; 1.49)	0.66 (0.27; 1.64)	0.68 (0.33; 1.40)	0.66 (0.40; 1.08)	0.87 (0.17; 4.30)	0
≥ 6 hours	0.93 (0.36; 2.40)	0.86 (0.26; 2.80)	0.47 (0.18; 1.24)	1.25 (0.58; 2.71)	3.69 (0.22; 60.80)	0
**Indicators of health status**						
Sickness absence (days/8 weeks)	0.98 (0.94; 1.02)	0.99 (0.94; 1.03)	**0.95 (0.92; 0.99)**	1.00 (0.97; 1.03)	1.28 (0.98; 1.67)	3
Depressive symptoms (CES-D scale)	**0.94 (0.92; 0.97)**	**0.89 (0.86; 0.93)**	**0.89 (0.87; 0.92)**	**0.93 (0.91; 0.95)**	0.96 (0.90; 1.03)	10

^*^Odds ratio (OR) and 95% confidence interval (95% CI) from logistic regression analyses. The full models are presented. All variables were entered simultaneously to analyze the independent association of each variable with self-sufficiency. Note: bold numbers indicate a statistically significant (*p*<0.05) association

^a^ Intermediate vocational education consists of four levels: 1=assistant training; 2=basic vocational training; 3=vocational training; 4=middle-management training

^b^ Binge drinking was defined as consuming five or more alcoholic drinks on one occasion

**Table 5:** Results of the longitudinal ordinal regression analyses evaluating associations of contextual factors and indicators of health status with overall self-sufficiency (*n* = 200)

	Univariable model of self-sufficiency^a^	Multivariable model of self-sufficiency^b^
	OR (95%CI)*	OR (95%CI)*
**Contextual factors**		
*Socio-demographics and context*		
Age (in years)	**0.80 (0.69; 0.93)**	0.96 (0.78; 1.18)
Gender		
Male	Ref.	Ref.
Female	0.73 (0.38; 1.38)	1.09 (0.48; 2.49)
Intermediate vocational education^c^		
Level 1–3	Ref.	Ref.
Level 4	0.78 (0.39; 1.57)	1.23 (0.52; 2.92)
Ethnic background		
Dutch	Ref.	Ref.
Non-Dutch	0.95 (0.49; 1.86)	0.80 (0.34; 1.87)
Living situation		
With caretaker	Ref.	Ref.
Not with caretaker	**0.21 (0.07; 0.61)**	0.25 (0.05; 1.36)
Perceived school performance		
> Average	Ref.	Ref.
≤ Average	0.59 (0.32; 1.08)	0.88 (0.43; 1.83)
*Risk behaviors*		
Current smoking		
No	Ref.	Ref.
Yes	0.53 (0.28; 1.01)	0.90 (0.39; 2.07)
Binge drinking^d^		
0 times/4 weeks	Ref.	Ref.
≥ 1 time/4 weeks	0.71 (0.41; 1.24)	0.67 (0.34; 1.35)
Cannabis use		
0 times/4 weeks	Ref.	Ref.
≥ 1 time/4 weeks	**0.25 (0.10; 0.60)**	0.59 (0.18; 1.93)
Criminal behavior		
0 times/year	Ref.	Ref.
≥ 1 time/year	0.50 (0.16; 1.62)	0.86 (0.21; 3.57)
Truancy		
0 h	Ref.	Ref.
1–5 h	1.10 (0.60; 2.02)	1.66 (0.77; 3.57)
≥ 6 h	0.36 (0.10; 1.26)	0.67 (0.14; 3.10)
**Indicators of health status**		
Sickness absence (days/ 8 weeks)	**0.93 (0.89; 0.98)**	0.98 (0.93; 1.04)
Depressive symptoms (CES-D scale)	**0.90 (0.87; 0.93)**	**0.90 (0.86; 0.93)**

Note: Self-sufficiency was entered as an ordinal variable ranging from self-sufficient on all life-domains, self-sufficient on ten life-domains, self-sufficient on nine life-domains and self-sufficient on eight or less life-domains. Odds ratios represent the odds for a participant to be allocated within a higher self-sufficiency category if they would have scored one point higher on the predictor variable. Bold numbers indicate a statistically significant (*p* < 0.05) association

^*^Odds ratio (OR) and 95% confidence interval (95% CI) from ordinal regression analyses

^a^ The predictor variables were entered separately in the univariable model

^b^ The predictor variables were entered simultaneously in the multivariable model

^c^ Intermediate vocational education consists of four levels: 1 = assistant training; 2 = basic vocational training; 3 = vocational training; 4 = middle-management training

^d^ Binge drinking was defined as consuming five or more alcoholic drinks on one occasion

Odds ratios (ORs) and 95% confidence intervals (CIs) were estimated. For the ordinal regression models, we present the univariable and multivariable models. The estimated ORs represent the odds for a student to be allocated within a higher self-sufficiency category if they would have scored one point higher on a predictor variable [30].

We considered a *p*-value of 0.05 or lower to be statistically significant. All analyses were performed using SPSS version 25 (IBM Corp. Released 2017. IBM SPSS Statistics for Windows, Version 25.0. Armonk, NY: IBM Corp.).

## Results

### Participant characteristics

Participants were on average 18.6 years old (SD = 2.04, with a minimum age of 16 years and a maximum age of 26 years); 73.6% were female and 60.6% were classified as Dutch (Table 1). Results of the lost to follow-up analysis showed that participants lost to follow-up were more often male (*p* < 0.05), were lower educated (*p* < 0.001) and more often had a non-Dutch ethnic background (*p* < 0.001) than participants included at both time-points (see additional file 3: Table C1).

The judicial life-domain had the highest number of self-sufficient participants at baseline (97.8%) and at follow-up (98.9). The three life-domains with the lowest number of self-sufficient participants were ‘finances’ (at baseline 73.5%, at follow-up 72.2%), ‘mental health’ (at baseline 60.0%, at follow-up 53.9%), and ‘physical health’ (at baseline 71.1%, at follow-up 64.4%) (Table 2).

### Results of the regression analyses

Table 3 shows the associations of contextual factors and indicators of health status with overall self-sufficiency at baseline, as assessed with ordinal regression analyses. In de multivariable model, young adults using cannabis were at risk of having a lower overall self-sufficiency category (OR = 0.57, 95% CI = 0.33 to 0.99), as were young adults with more sick days from school (OR = 0.94, 95% CI = 0.91 to 0.98), and young adults with a higher score on the depressive symptoms scale (OR = 0.87, 95% CI = 0.85 to 0.89).

Tables 4 presents the results of the associations of contextual factors and indicators of health status with the separate self-sufficiency life-domains at baseline, assessed with logistic regression analyses. An increase in sick days was associated with lower odds of being self-sufficient on three life-domains, ORs varied from 0.92 to 0.95. An increase on the scale of depressive symptoms was associated with lower odds of being self-sufficient on ten life-domains, ORs varied from 0.81 to 0.96.

Table 5 shows the results of the associations of contextual factors and health status at baseline with overall self-sufficiency at follow-up, assessed with ordinal regression analyses. In de multivariable model, young adults with a higher score on the depressive symptoms scale were at risk of having a lower overall self-sufficiency category (OR = 0.90, 95% CI = 0.86 to 0.93).

## Discussion

This study investigated the association of young adults’ self-sufficiency to function in daily life with contextual factors and indicators of health status. Our results suggest that the life-domains ‘finances’, ‘mental health’, and ‘physical health’ were most often reported as a problem area regarding self-sufficiency among young adults. Furthermore, young adults reporting more sick days from school or higher depressive symptom levels were less likely to be self-sufficient overall and on several life-domains.

Our finding that a large percentage of young adults participating in our study seemed to face challenges in functioning in the life-domains of ‘finances’, ‘mental health’, and ‘physical health’ was partly in line with a previous study. Bannink et al. [21] studied the same eleven life-domains of self-sufficiency among a comparable sample of intermediate vocational education students aged 18.3 years on average. Similar to our study, they reported that the life-domains ‘finances’ and ‘mental health’ were the most challenging areas. However, the second largest problem area in their study was ‘domestic relations’, while in our study, this was ‘physical health’ (i.e. physical self-sufficiency regarding sickness and the ability to deal with the sickness). Furthermore, their study showed overall fewer problems in being self-sufficient on all life-domains. These differences could be explained by an overrepresentation of young adults who display extensive sickness absence as a result of our sample selection, in which we purposely aimed to select more students with extensive sickness absence.

Overall, especially indicators of health status (i.e. sickness absence and depressive symptoms) were related to diminished overall self-sufficiency and self-sufficiency on specific life-domains, for instance ‘daytime activities’ and ‘social network’. These findings concur with previous research where mental disorders were found to affect daily functioning by limiting personal, social, and work life [31]. And where depressive symptom levels among adolescents were inversely related to life satisfaction, and academic and emotional self-efficacy [32, 33]. Finally, absence from school, either due to sickness or truancy, was found to be related to low levels of academic achievements and antisocial or risky behaviors, such as ineffective coping [34, 35].

Cannabis use was related to diminished overall self-sufficiency in the ordinal regression analyses, but not in the logistic regression analyses for the separate life-domains. This difference may be due to the fact that cannabis users had the likelihood of falling especially into the group that was self-sufficient on eight or fewer life-domains on the ordinal variable (data are not shown). This suggests that cannabis users are more likely to have trouble with self-sufficiency on multiple life-domains simultaneously.

The finding that young adults who do not live with a caretaker are less self-sufficient on the life-domains ‘finances’ and ‘housing’ suggests that specific attention should be given to young adults’ financial situation and future housing situation, especially for those who are on the verge of moving out of their caretakers’ house. Parents, school staff and youth health care professionals should be encouraged to address this, for example in the form of parental role-modeling or parental teaching and by communication between parents and their child about work [36, 37].

Our findings underline the importance of early promotion of self-sufficiency, preferably before the transition from adolescence to young adulthood has begun. In this regard, it is recommended to stimulate their social-emotional competencies through social and emotional learning programs [38] and to use resilience-focused school-based interventions [39]. These resilience-focused interventions focus on resilience protective factors (e.g. personal strengths and qualities of community environments) that enable an individual to thrive and to overcome disadvantage. Also, sickness absence and depressive symptoms appear to be risk factors associated with diminished self-sufficiency and should therefore be monitored and addressed when empowering young adults in their functioning in daily life.

This study has some limitations that warrant consideration when interpreting the results. First, some factors that are possibly related to self-sufficiency were not assessed. For instance, income as a possible factor affecting (financial) self-sufficiency or dietary and sedentary behaviors as possible factors affecting (mental) health [40–42]. Second, this study was exploratory in the sense that we used a large set of predictor variables to test for possible associations with self-sufficiency. If we would apply the Bonferroni correction for multiple testing on both the ordinal regression analyses (corrected significance level is 0.05/13 = 0.004), similar results would be obtained, except for the non-significant association of cannabis use and self-sufficiency after correction. Third, the questionnaires were completed at two time points, therefore we could not infer causality. Also, a relatively large number of participants were lost to follow-up, which could have led to power problems in detecting significant associations at follow-up. A large study with multiple follow-up measures is recommended to gain insight into the direction of the associations. Lastly, our study was done in a population of students attending intermediate vocational education, which is a relatively low level of education [17]; also, there was an overrepresentation of students with extensive sickness absence from school; therefore, we recommend to repeat this study in large varied samples in other countries and settings.

## Conclusions

This study assessed the association of young adults’ self-sufficiency to function in daily life with contextual factors and indicators of health status. Several factors were associated with worse self-sufficiency, especially indicators of health status in the form of sick days from school and depressive symptoms. Our findings underline the importance of early promotion of self-sufficiency, preferably before the transition from adolescence to young adulthood has begun. It is recommended to stimulate adolescents’ and young adults’ social-emotional competencies, and to address sickness absence and depressive symptoms.

## Supplementary information

**Additional file 1: Table A1.** Description of life-domains derived from the Dutch self-sufficiency matrix.

**Additional file 2: Figure B1.** Studied factors incorporated in the International Classification of Functioning, Disability and Health (ICF) of the WHO.

**Additional file 3: Table C1.** Lost to follow-up analyses on socio-demographic characteristics (*N* = 755).

## Data Availability

Data are available upon reasonable request by contacting the corresponding author Hein Raat (h.raat@erasmusmc.nl).

## Abbreviations

ICF: International classification of functioning, disability, and health
MASS: Medical advice for sick-reported students
IVE: Intermediate vocational education
SSM-D: The Dutch version of the self-sufficiency matrix
CES-D: Center for epidemiologic studies depression scale
OR: Odds ratio
CI: Confidence interval
SD: Standard deviation
