# Supplementary material for: Young adults’ self-sufficiency in daily life: the relationship with contextual factors and health indicators
Source: BMC Psychol. 2020 Aug 28;8:89. doi: 10.1186/s40359-020-00434-0 (PMC7456010; doi:10.1186/s40359-020-00434-0)
Supplement: Supplementary file 1 — Additional file 1: Table A1. Description of life-domains derived from the Dutch self-sufficiency matrix. [file 40359_2020_434_MOESM1_ESM.docx]

Additional file 1

| **Table A1.** Description of life-domains derived from the Dutch self-sufficiency matrix | |
| --- | --- |
| **Life-domain** | **Description** |
| Finances | *Think of:* having the money to make ends meet |
| Daytime activities | *Think of:* attending a course or education, go to the classes |
| Housing | *Think of:* having a fixed and safe place to live |
| Domestic relations | *Think of*: trusting each other, supporting each other |
| Mental health | *Think of:* feeling good and being happy |
| Physical health | *Think of:* having a disease or disability and being able to deal with that |
| Addiction | *Think of:* quitting with gaming, alcohol or cannabis |
| Activities daily life | *Think of:* eating, washing, getting dressed, grocery shopping |
| Social network | *Think of:* support from family and friends |
| Community participation | *Think of:* leisure time activities |
| Judicial | *Think of:* contact with police or having a criminal record |
