# Supplementary material for: Young adults’ self-sufficiency in daily life: the relationship with contextual factors and health indicators
Source: BMC Psychol. 2020 Aug 28;8:89. doi: 10.1186/s40359-020-00434-0 (PMC7456010; doi:10.1186/s40359-020-00434-0)
Supplement: Supplementary file 2 — Additional file 2: Figure B1. Studied factors incorporated in the International Classification of Functioning, Disability and Health (ICF) of the WHO. [file 40359_2020_434_MOESM2_ESM.docx]

Additional file 2

| **Health condition**   - Sickness absence (number of sick days from school in the past eight weeks) - Depressive symptoms scale (Center for Epidemiologic Studies Depression scale (CES-D)) |
| --- |
|  |
| Self-sufficiency **(participation and activity)** |
|  |
| **Contextual factors (personal and environmental factors)**   - Socio-demographics and context (age, gender, vocational education level, ethnic background, living situation, and perceived school performance) - Risk behaviors (smoking, binge drinking, cannabis use, criminal behavior, truancy) |
| Figure B1. Studied factors incorporated in the International Classification of Functioning, Disability and Health (ICF) of the WHO. |
