# Supplementary material for: Young adults’ self-sufficiency in daily life: the relationship with contextual factors and health indicators
Source: BMC Psychol. 2020 Aug 28;8:89. doi: 10.1186/s40359-020-00434-0 (PMC7456010; doi:10.1186/s40359-020-00434-0)
Supplement: Supplementary file 3 — Additional file 3: Table C1. Lost to follow-up analyses on socio-demographic characteristics (N = 755). [file 40359_2020_434_MOESM3_ESM.docx]

Additional file 3

| **Table C1.** Lost to follow-up analyses on socio-demographic characteristics (N=755) | | | | |
| --- | --- | --- | --- | --- |
|  |  | Population for longitudinal analyses  n=200 | Lost to follow-up  n=555 | *p*-value |
| **Socio-demographic characteristics** |  |  |  |  |
| Age in years, mean (SD) | [3] | 18.5 (1.9) | 18.6 (2.1) | .711 |
| Gender, n female (%) | [2] | 157 (78.9) | 398 (71.7) | **.049** |
| Intermediate vocational education level, n level 4 (%) | [28] | 153 (80.1) | 325 (60.6) | **.000** |
| Ethnic background, n Dutch (%) | [9] | 151 (77.4) | 301 (54.6) | **.000** |

Note: [number of missing answers]. Bold numbers indicate statistical significance (p<0.05) between the population for the longitudinal analyses and lost to follow-up
